# Comparison between two cancer registry quality check systems: functional features and differences in an Italian network of cancer registries dataset

**DOI:** 10.3389/fonc.2023.1197942

**Published:** 2023-05-25

**Authors:** Giovanna Tagliabue, Viviana Perotti, Sabrina Fabiano, Andrea Tittarelli, Giulio Barigelletti, Paolo Contiero, Walter Mazzucco, Mario Fusco, Ettore Bidoli, Massimo Vicentini, Maria Teresa Pesce, Fabrizio Stracci, The Collaborative Working Group

**Affiliations:** ^1^ Cancer Registry Unit, Fondazione IRCCS Istituto Nazionale dei Tumori, Milan, Italy; ^2^ Environmental Epidemiology Unit, Fondazione IRCCS Istituto Nazionale dei Tumori, Milan, Italy; ^3^ Clinical Epidemiology Unit and Palermo Province Cancer Registry, University Hospital “P. Giaccone”, Palermo, Italy; ^4^ Department of Oncology and Public Health, Executive Board of the Italian Network of Cancer Registries (AIRTUM), Milan, Italy; ^5^ Cancer Registry Unit, ASL Napoli 3 Sud, Naples, Italy; ^6^ Cancer Epidemiology Unit, Centro di Riferimento Oncologico (CRO), IRCCS, Aviano, Italy; ^7^ Epidemiology Unit, Azienda Unità Sanitaria Locale - IRCCS di Reggio Emilia, Reggio Emilia, Italy; ^8^ Cancer Registry Unit, ASL Caserta, Caserta, Italy; ^9^ Umbria Regional Cancer Registry, Department of Medicine and Surgery, University of Perugia, Perugia, Italy

**Keywords:** data quality, population-based cancer registry, incidence, quality check systems, IARC, JRC-ENCR, cancer research

## Abstract

**Purpose:**

The aim of this study was to compare the functional characteristics of two computer-based systems for quality control of cancer registry data through analysis of their output differences.

**Methods:**

The study used cancer incidence data from 22 of the 49 registries of the Italian Network of Cancer Registries registered between 1986 and 2017. Two different data checking systems developed by the WHO International Agency for Research on Cancer (IARC) and the Joint Research Center (JRC) with the European Network of Cancer Registries (ENCR) and routinely used by registrars were used to check the quality of the data. The outputs generated by the two systems on the same dataset of each registry were analyzed and compared.

**Results:**

The study included a total of 1,305,689 cancer cases. The overall quality of the dataset was high, with 86% (81.7-94.1) microscopically verified cases and only 1.3% (0.03-3.06) cases with a diagnosis by death certificate only. The two check systems identified a low percentage of errors (JRC-ENCR 0.17% and IARC 0.003%) and about the same proportion of warnings (JRC-ENCR 2.79% and IARC 2.42%) in the dataset. Forty-two cases (2% of errors) and 7067 cases (11.5% of warnings) were identified by both systems in equivalent categories. 11.7% of warnings related to TNM staging were identified by the JRC-ENCR system only. The IARC system identified mainly incorrect combination of tumor grade and morphology (72.5% of warnings).

**Conclusion:**

Both systems apply checks on a common set of variables, but some variables are checked by only one of the systems (for example, checks on patient follow-up and tumor stage at diagnosis are included by the JRC-ENCR system only). Most errors and warnings were categorized differently by the two systems, but usually described the same issues, with warnings related to “morphology” (JRC-ENCR) and “histology” (IARC) being the most frequent. It is important to find the right balance between the need to maintain high standards of data quality and the workability of such systems in the daily routine of the cancer registry.

## 1 Introduction

One of the main objectives of population-based cancer registries is to collect complete and accurate data on cancers diagnosed in the population under registration. Data quality is an important issue in cancer registration because incomplete or poor-quality data generate flawed results.

Each cancer registry uses its own, internal rules for cancer coding and registration, as well as common rules developed and used by both the corresponding national registration network and the international registration networks, such as the European Network of Cancer Registries (ENCR) or the International Agency for Research on Cancer and the International Association of Cancer Registries (IARC/IACR). Registrars are encouraged to attend proposed training courses: for example, the North American Association of Central Cancer Registries (NAACCR) offers professional qualification and refresher courses, so that cancer registration is done in the most standardized way possible, with little variation due to personal interpretation or lack of up-to-date information.

In recent years, some registries have been using electronic health records for incidence calculation. Created for administrative purposes, electronic health records are timely and inexpensive but do not provide the same degree of clinical detail as medical records. They can be very useful, however, to improve the completeness and quality of cancer incidence data (e.g., pharmaceutical databases for drug treatment of cancer patients).

Data quality checks can be done at different points in time.

The NAACCR network in the US provides registries with a program that checks data quality at the time of data entry but also on already entered records (GenEDITS Plus) (1).

In Europe, IARC (2) and the European Commission Joint Research Center (JRC) in collaboration with the ENCR (3, 4) have made available to cancer registry operators two computer-based edit check systems: the IARC/IACR CHECK program and the JRC-ENCR quality check software. Both systems automatically check the quality of the data produced by the registries, leading to the definition of high-quality datasets standardized according to international criteria (3, 5, 6).

Each of these check systems has its own characteristics: both analyze common as well as system-specific variables and identify errors and deficiencies that, if corrected, will improve the quality of the generated data.

Every five years, IARC calls on cancer registries around the world to send in their data, so it can update the database it maintains and uses to monitor cancer. Based on the collective registry data IARC publishes the volume *Cancer Incidence in Five Continents*, an “invaluable source of information about the global burden and distribution of cancer” (2). In conjunction with this call, the Joint Research Center (JRC) of ENCR has also requested the submission of incidence databases from European registries, to build a large European database in the framework of the European Cancer Information System (7). To produce valid results, the submitted data must be comparable with each other, as complete as possible, and of good quality.

The aims of this study were two: to perform a quality evaluation of the data submitted to these international calls, and to compare the functional characteristics of the two most used systems to check the accuracy of cancer registry data.

The datasets of each registry participating in the study were checked with both systems. Outputs were compared to identify the characteristics and differences detected by each system in an effort to improve the quality of the recorded data and assess the functionality of each check system.

## 2 Materials and methods

### 2.1 Data sources

Twenty-two Italian population-based cancer registries affiliated with the Italian Network of Cancer Registries (AIRTUM) (8) participated in the study. The analyzed data spanned from 1986 to 2017, depending on the incidence periods recorded by each registry.

AIRTUM coordinates the national network of general and specialized (pediatric and pancreatic cancer) population-based cancer registries. It designs and conducts collaborative descriptive studies and research activities related to cancer epidemiology in Italy.

Italian cancer registries routinely collect data on incident cancer cases among all residents in the covered area through clinical records, regional mortality files, pathology files, pharmacology files, laboratory databases and hospital discharge databases (electronic health records). The data are collected by trained registrars according to established abstracting rules and standardized manuals such as the *International Classification of Diseases for Oncology*, third edition (ICD-O-3) and the *TNM Staging Manual* (9, 10). For the present study all registries sent in data on all primary tumors including data (if collected) of non-malignant tumors of the central nervous system and urinary bladder.

The registrars use all available pathologic and clinical information to document the date of diagnosis, ICD-O-3 cancer site (topography), histology (morphology), tumor behavior, stage, cancer-specific characteristics (e.g., human epidermal growth factor receptor-2, prostate-specific antigen, Gleason score), demographics and follow-up for vital status.

Data are structured as one record per person per cancer: persons with multiple cancers have multiple records.

### 2.2 Data quality

Measurement of the quality of registry data is based on four parameters: comparability, completeness, accuracy and timeliness (11). Our analysis was mainly focused on the accuracy of cancer registry data.

### 2.3 Quality checks

The data were processed using two computer-based data-checking systems developed to assess the quality of population-based cancer registry data.

The IARC/IACR CHECK program, produced by the World Health Organization, is freely available (5). It was created to assess the quality of data provided by registries from worldwide countries for the publication of *Cancer Incidence in Five Continents*. It validates code assignment (sex, incidence and birth date, ICD-O-3 topography, morphology and behavior) and checks the consistency between data items (age versus birth and incidence dates, chronology between birth and incidence dates, sex versus site, sex versus histology, age versus site, age versus histology, site versus histology, basis of diagnosis versus histology).

The JRC-ENCR quality check software (3) is produced by the JRC in collaboration with ENCR and is freely available for the quality control of cancer registry data. It checks for consistency within variables (patient record format, date of cancer incidence, basis of diagnosis, tumor characteristics and stage at diagnosis and patient follow-up) and consistency between variables (coherence between date of birth, date of incidence and date of last known vital status; consistency between age, tumor topography and morphology; consistency between basis of diagnosis, tumor morphology and behavior; consistency between tumor morphology and grade, between topography and laterality, between topography and morphology). Lastly, it offers the possibility of checking the consistency of vital status and autopsy, autopsy and basis of diagnosis, survival, date of incidence and follow-up.

The number of variables used in the checks is greater in the JRC-ENCR than in the IARC check system. For example, JRC-ENCR evaluates variables such as stage at cancer diagnosis, vital status and patient follow-up.

The two systems generate two types of indicators from the checked datasets: errors and warnings. These are specified with short labels that may differ depending on the system used (see **Supplementary Materials**).

Errors are defined as unacceptable values of variables or unacceptable combinations of variables (impossible code, impossible code combination, missing variable, wrong format or value of variable out of range), while warnings pertain to unusual codes or unlikely code combinations (possible but very rare code or possible but very rare code combination), which may, however, be accepted after specific verification.

Data analysis on quality checks performed by the JRC-ENCR and IARC systems for multiple primaries was not part of this study.

## 3 Results

### 3.1 AIRTUM cancer registries

**Table 1** lists the contributing cancer registries and the number of cases provided by each registry. We analyzed 22 Italian population-based cancer registries for a total of 1,305,689 cases with different incidence periods (spanning from 1986 to 2017) depending on the registry.

**Table 1:** The 22 Italian cancer registries participating in the study.

Cancer registry	Number of cases	Years of incidence
Avellino	16566	2010-2015
Benevento	13829	2010-2016
Bolzano	67045	1995-2017
Caserta	47509	2008-2016
Catania-Messina-Enna	174866	2003-2017
Friuli Venezia Giulia	60054	2013-2017
Genova	231368	1986-2016
Napoli 1	41128	2010-2015
Napoli 2	38862	2010-2016
Napoli 3	34231	2013-2017
Nuoro	4568	2013-2015
Palermo	106923	2003-2017
Pavia	63232	2003-2017
Ragusa-Caltanissetta	18980	2013-2017
Salerno	45796	2010-2016
Sassari	10695	2012-2015
Siracusa	44040	1999-2017
Sondrio	5702	2013-2015
Trapani-Agrigento	34813	2002-2013
Trento	84163	1995-2017
Umbria	156914	1994-2017
Valle d’Aosta	4405	2013-2017
**Total**	**1305689**	

### 3.2 Data quality checks

The median percentages of DCO cases (cancer with a diagnosis by death certificate only) and microscopically verified cases were 1.2% (range 0.03 to 3.06) for males and 1.4% (range 0.03 to 3.2) for females and 86.3% (range 81.7 to 93.9) for males and 87.3% (range 82.7 to 94.1) for females (data not shown), respectively. Standardized incidence and mortality rates, included temporal trends, where computed (data not shown); the integrated interpretation of these indicators add evidence of the good quality of cancer data of Italian registries.

In this analysis, only variables that presented problems are discussed. For the complete list of variables used by the two check systems, see the **Supplementary Materials**.

### 3.3 General analysis

#### 3.3.1 Errors

Both systems detected some errors in the checked cases. In the 1,305,689 cases checked, the JRC-ENCR system detected 2,248 errors (0.17%) and the IARC system 45 errors (0.003%). **Table 2** lists the detected errors by type.

**Table 2:** Types of errors reported by the check systems (common types of errors between the two systems are aligned).

JRC-ENCR		n	%	IARC	n	%
E-CoDV	Date of last known vital status not valid	13	0.58			
E-FORM	Format error	433	19.26			
E-MISS	Value missing	60	2.67			
E-OUTR	Value out of range*	1741	77.45	ICD-O-3 (Topography)*	22	48.89
				ICD-O-3 (Morphology)*	22	48.89
E-SETO	Sex + Topography not valid	1	0.04	Sex/site combination	1	2.22
**Total**		2248	100		45	100

*Topography and Morphology IARC errors are included in E-OUTR category of JRC-ENCR system

The proportion of true errors identified by the JRC-ENCR system was 98%, whereas this proportion was 2.2% with the IARC check system. Both system identified the same false errors (n=44).

#### 3.3.2 Warnings

Among the 1,305,689 checked cases, the JRC-ENCR system reported 36,534 warnings (2.8%) and the IARC system 31,700 (2.4%) (**Table 3**).

**Table 3:** Types of warnings reported by the systems (common types of warnings between the two systems are aligned).

JRC-ENCR		n	%	IARC	n	%
W-AGMT	Unlikely age group + tumor type	632	1.73	Age/site/histology	490	1.55
W-BDMO	Morphology too specific	11711	32.06	Basis/histology	5895	18.60
				Behavior/histology	89	0.28
W-BDMS	Morphology not specific enough	10414	28.50			
W-BDMU	BoD + Morpho/Behavior	811	2.22			
W-BDpM	BoD + pM not valid	724	1.98			
W-BDpN	BoD + pN not valid	1119	3.06			
W-BDpT	BoD +pT not valid	1444	3.95			
W-BTNM	Behavior + TNM not valid	170	0.47			
W-MISS	A non-compulsory variables missing	23	0.06			
W-MOGR	Morphology + grade not valid	2906	7.95	Grade/morphology	22994	72.54
W-MOBE	Morphology + Behavior not valid	73	0.20			
W-MOTO	Morphology + Topo not valid	6483	17.75	Histology/site	2200	6.94
W-SEMO	Sex + Morphology not valid	12	0.03	Sex/histology	32	0.10
W-TNMS	Topo + TNM edition + T,N,M + Pathologic stage not valid	6	0.02			
W-UNKN	Unknown code found	6	0.02			
**Total**		36534	100		31700	100

BoD, basis of diagnosis.

The distribution of warnings by registry differed between the two check systems, from a maximum of 10.93% to a minimum of 0.38% with the JRC-ENCR system and from a maximum of 9.23% to a minimum of 0.12% with the IARC system (data not shown).

### 3.4 Comparison of JRC-ENCR and IARC check systems

This part of the analysis concerns comparisons between errors and warnings identified by the JRC-ENCR and IARC systems. A case may present one or more problems (errors and/or warnings) simultaneously, which may either be reported by both systems or by one of them only. When an error or warning detected by both check systems is identified, it means it has been categorized in the same way by both systems. The IARC check system detected 45 errors in the analyzed registry data; the errors categorized in the same way by the JRC-ENCR system were 42 (**Table 4**).

**Table 4:** Errors reported by both or one of the systems.

Errors	n	%
JRC-ENCR and IARC	42	1.87
Only JRC-ENCR	2206	98.00
Only IARC	3	0.13
**Total**	**2251**	100

In the case series examined, 29,467 warnings (48.17% of total warnings) were detected only by the JRC-ENCR system and 24,633 (40.27% of total warnings) only by the IARC system, while 7,067 warnings (11.55% of total warnings) were detected and categorized in the same way by both systems (**Table 5**). The differences can be attributed to the different number of variables considered by the two check systems: the IARC system considered 10 variables and the JRC-ENCR system 39.

**Table 5:** Warnings reported by both or one of the systems.

Warning	n	%
JRC-ENCR and IARC	7067	11.55
Only JRC-ENCR	29467	48.17
Only IARC	24633	40.27
**Total**	**61167**	100

The types of warnings reported by the JRC-ENCR system only are presented in **Table 6**, while **Table 7** lists the types of warnings reported by the IARC system only.

**Table 6:** Warnings reported by JRC-ENCR only.

JRC-ENCR	warnings	n	%
W-BDMS	Morphology not specific enough	10414	35.34
W-BDMO	Morphology too specific	6202	21.05
W-MOTO	Morphology + Topography not valid	6035	20.48
W-MOGR	Morphology + Grade not valid	2269	7.70
W-BDpT	BoD + pT not valid	1444	4.90
W-BDpN	BoD + pN not valid	1119	3.80
W-BDMU	BoD + Morpho/Behavior	811	2.75
W-BDpM	BoD + pM not valid	724	2.46
W-AGMT	Unlikely Age group + Tumor type	171	0.58
W-BTNM	Behavior + TNM not valid	170	0.58
W-MOBE	Morphology + Behavior not valid	73	0.25
W-MISS	A compulsory variable is missing	23	0.08
W-UNKN	Unknown code found	6	0.02
W-TNMS	Topo + TNM edition + T,N,M + Pathologic stage not valid	6	0.02
**Total**		**29467**	**100**

BoD, basis of diagnosis.

**Table 7:** Warnings reported by IARC only.

IARC warning	n	%
Grade/morphology	22357	90.76
Histology/site	1752	7.11
BoD/histology	386	1.57
Behavior/histology	89	0.36
Age/site/histology	29	0.12
Sex/histology	20	0.08
**Total**	**24633**	**100**

BoD, basis of diagnosis.

**Table 8** shows some of the most common combinations of topographies and morphologies flagged as warnings by the two check systems, listed by number and type. The JRC-ENCR system specifically flags the coding of morphologies of the hematopoietic system in tumors arising at sites other than bone marrow (429 warnings and 932 warnings depending on the morphology considered). The largest number of warnings with the IARC system (585) concerns certain morphologies of ovarian pertinence coded in tumors arising in the pancreas, peritoneum, and uterine cervix and body. The differences between the systems can be attributed to the different criteria defining the morphology-site combination.

**Table 8:** Most common examples of morphology and site combinations (ICD-O-3 codes) reported as warnings.

Topography*	Morphology	No. of warnings	
		JRC-ENCR	IARC
C42/C77	8000	314	0
C38.1-C38.3/C41/C48/C71/C77	Epithelial morphology	85	31
≠ C34	8012 or 8041-8045	8	299
C44	8098	141	0
≠ C67	8120 or 8130	101	35
C20.9, C21.1, C21.8-C21.9	8124	91	0
C11, C21-C24, C30-C34, C56	8144	6	72
C22.0 or C24-C25	8160-8162	206	1
C50	8401	0	216
C25, C48, C53-C55	8441, 8460, 8470-8471	24	585
C73.9	8510	227	0
≠ C30.0, C44, C51, C60, C63.2, C69.0, C80	8770-8772	161	23
C74.9	8370, 8700, 9490, 9500	132	0
C71-C72	9530-9539	106	0
C80.9	9590-9591 or 9699	233	0
C42.1	9220, 9731, 9761, 9930	446	1
≠ C42.1	9732	429	57
C42.0 or C42.2 or C42.4	9820-9823, 9860-9863	932	0
C42.0 or C42.4	9590-9591, 9650-9653, 9670-9673, 9680, 9690	299	0
C64.9	8120, 8130, 8210-8211	70	0
C07-C09, C12-C17, C30-C33, C38, C42-C54, C61-C68, C71-C77	8240, 8243-8246, 8249	37	242

*See **Table S5**.

## 4 Discussion

There is an obvious need to control the quality of data produced by cancer registries. Quality control takes place when data are used to carry out research, for example a survival study (9); to manage large databases of registry data (10); or to evaluate the performance of the registry itself (11). The present analysis addresses the quality control of population-based registry data by measuring the efficacy of two computer-based check systems. To our knowledge, this is the first published analysis of its kind.

### 4.1 Errors

The JRC-ENCR software tends to find a greater number of errors because, unlike the IARC system, it includes the evaluation of variables related to patient follow-up, vital status and TNM staging.

Both systems report errors such as the use of incorrect ICD-O-3 topography codes (for example, C22.9, C26.1, C45.0, liver unspecified, spleen, mesothelioma of pleura; all these are ICD-10 codes) (12). The IARC program also reports on morphologies it fails to recognize, for example those coded 8741, 8349, 8509 and 8348, which are new morphology codes included in the revised version of ICD-O-3 (13) and already in use by registries. This issue will be easily solved with updated checking algorithms.

### 4.2 Warnings

#### 4.2.1 Morphology and topography

Both systems flag unusual combinations of morphology and topography, but use different criteria in the selection of such combination.

According to Berg, tumors with a primitive or mixed cell type may develop in any organ. They may arise from pluripotent stem cells remaining in the organ or by dedifferentiation, and this may explain why almost any type of cancer can be found in almost any site upon occasion (14).

The JRC-ENCR check system flags up certain combinations of morphology and topography that the IARC system does not identify as incompatible. For example, it rejects the combination of morphology 8000 (neoplasm) with topographies C42.0, C42.1 and C77 (blood, bone marrow and lymph node); it accepts morphology 8098 (adenoid basal carcinoma) only in the cervix uteri, while the IARC system accepts it also in C44 (skin); it accepts morphology 8124 (cloacogenic carcinoma) only for tumors in C21.2 (cloacogenic zone), whereas the IARC system accepts it at other sites of the gastrointestinal tract as well (C20.9 rectum, C21.1 anal canal, C21.8 overlapping lesion of rectum, anus and anal canal). Cloacogenic carcinoma, also called basaloid carcinoma, is an entity originating from the anal transitional epithelium. It is debated whether this neoplasm should be considered a separate entity from squamous cell carcinoma of the anal canal, given the differences in cells of origin, proteomic signatures and survival rates (15), or be classified as a carcinoma of squamous cell nature but manifesting a tendency toward glandular differentiation similar to that sometimes seen in tumors of the oral cavity, larynx or esophagus, currently designated as basaloid carcinomas (16). The specific expression of several types of cell keratins in the anal transitional zone is also found in epithelium of other squamocolumnar junctions such as the esophagogastric and endo-exocervical junctions (17, 18). The literature reports very rare cases of basaloid cell carcinoma arising in the colon and rectum (19).

The morphology code 8510 (medullary carcinoma) for tumors arising in the thyroid gland is accepted by the IARC but not the JRC-ENCR system. The JRC-ENCR system instead accepts code 8345 (medullary carcinoma with amyloid stroma) for thyroid cancer, reserving 8510 for cancers arising in breast, stomach and colon. This is justified by the fact that, despite some common morphologic features (lymphocytic infiltration, poorly differentiated cells), they are distinct entities. Medullary thyroid carcinoma is a neuroendocrine malignancy originating from parafollicular cells (C cells), whereas medullary carcinoma arising in other organs such as breast, stomach or colon is a very uncommon cancer (less than 5% of breast cancers and 0.05% of colon cancers) with neuroendocrine-like features, poorly differentiated aspects, microsatellite instability, lymphocytic infiltration and specific molecular characteristics (20). The American Network of SEER registries accepts both codes for thyroid carcinoma with medullary histology (21). The IARC system classifies medullary carcinoma as “not site-specific carcinoma” and therefore accepts it for cancers arising at any site except bone, connective tissue and nervous system (C40-C42, C47, C48, C49, C70, C71, C72, C77).

The morphology codes 8370, 8700, 9490 and 9500 (adrenal cortical carcinoma, pheochromocytoma, ganglioneuroma, neuroblastoma) are not accepted by the JRC-ENCR system at the generic site C74.9 (adrenal gland NOS), but only at specific subsites of the adrenal gland such as C74.0 (cortex of adrenal gland) or C74.1 (medulla of adrenal gland), according to the specific morphology. Neuroblastoma is due to differentiation arrest of the neural-crest-derived sympathoadrenal lineage. The sympathoadrenal lineage is derived from neural crest cells that emigrate from the dorsal neural tube and migrate to distant sites during the early stages of embryogenesis (22). Clinically, neuroblastoma manifests as a primary tumor anywhere along the sympathetic nervous system, with >50% occurring in the adrenal medulla (C74.1) (23). The site of origin is therefore C74.1 (medulla of adrenal gland), as correctly indicated by the JRC-ENCR system. The IARC system, however, accepts coding of this morphology also at the generic site C74.9. There is a plausible reason for this: the IARC system has a global distribution, and there are geographic areas where it is difficult to obtain the information needed for complete cancer incidence estimation, so all the collected information is used, even if it shows a lesser degree of accuracy (24).

Another difference is the use of code C80.9 (unknown primary site) in combination with hematopoietic morphologies (9590-9597 or 9699), which is accepted by the IARC system but not the JRC-ENCR system. The consistency check between topography and morphology brings to the fore two types of issues: the possible registration of an extranodal lymphoma whose precise organ of origin is unknown (the ICD-O-3 rule is to code lymphoma to C80.9, unknown primary site, if it is suspected to be extranodal and no site of origin is indicated) and the use of the code for unknown site rather than lymph node for a lymphoma of nodal origin. The JRC-ENCR system requires checking of all lymphoma cases coded with this topography, whereas the IARC system accepts any morphology of lymphoma at any site of origin because lymphomas are considered tumors with a non-specific site profile.

A further example involves morphology codes 8120 (transitional cell carcinoma) and 8130 (papillary transitional cell carcinoma), which according to the JRC-ENCR system are compatible with just a few sites of tumor origin (C56 ovary and C65-C68 renal pelvis, ureter, bladder, other urinary organs), while the IARC system accepts them for many other topographies (C11 nasopharynx; C14 other and ill-defined sites in lip, oral cavity and pharynx; C20 rectum, C21 anus and anal canal, C26 intestinal tract NOS; C30 nasal cavity and middle ear; C31 accessory sinuses; C53 cervix uteri; C61 prostate; C64 kidney). For tumors arising in the nasal cavity and accessory sinuses, the JRC-ENCR system accepts the morphology code 8121, Schneiderian (cylindrical [transitional] cell) carcinoma. Schneiderian carcinoma is a typical cancer of the nasal cavity and sinuses and is closely related to non-keratinizing squamous cell carcinoma. A typical feature is lack of maturation in the epithelial nests as in transitional cell carcinoma of the urinary tract, which this tumor subtype resembles.

Certain combinations of tumor morphology and topography are accepted by the JRC-ENCR system but trigger a warning from the IARC system. For example, morphology code 8401 (apocrine adenocarcinoma) is accepted for breast cancer by the JRC-ENCR system, while the IARC system accepts only C00 lip, C44 skin, C51 vulva, C60 penis, C63.2 scrotum NOS, C76 other and ill-defined sites as possible sites.

The IARC system reports tumors with a site-specific profile, but in most cases an unlikely combination between site and morphology according to IARC is accepted by JRC-ENCR. For example, adenocarcinoma, intestinal type (8144/3) is accepted for a larger number of sites by the JRC-ENCR than the IARC system.

Another difference in the selection of warnings concerns the use of morphology codes 8012 (large cell carcinoma NOS) or 8041-8045 (small cell carcinoma, oat cell carcinoma, small cell carcinoma fusiform cell, small cell carcinoma intermediate cell and combined small cell carcinoma) for tumors in sites other than the lung. Both systems limit the use of these morphologies to cancers arising in the respiratory system, but while the JRC-ENCR system considers coding these morphologies unusual only in tumors arising in C38, C40-C42, C47, C48.0, C49, C70-C72 and C77 (pleura, bone, joints and articular cartilage; peripheral nerves and autonomic nervous system; retroperitoneum and peritoneum; connective, subcutaneous and other soft tissues; meninges, brain and spinal cord, cranial nerves and other parts of the central nervous system; lymph nodes), the IARC system uses stricter limits and allows these morphologies only in cancers of the lung, ill-defined sites of the respiratory system, and intrathoracic organs (C34, C39.8, C39.9, C76.1 thorax, C76.7 other ill-defined sites, C76.8 overlapping lesion of ill-defined sites) in addition to unknown primary sites (C80.9). This results in the generation of a much greater number of warnings by the IARC system than the JRC-ENCR system.

The same applies to the use of morphology codes 8441 (serous cystadenocarcinoma), 8460 (papillary serous cystadenocarcinoma), 8470 and 8471 (mucinous cystadenocarcinoma and papillary mucinous cystadenocarcinoma) for cancers arising at sites C25 (pancreas), C48 (peritoneum and retroperitoneum) and C53, C54, C55 (cervix uteri, corpus uteri, uterus NOS). The IARC system accepts these morphologies only at the following sites: C56 (ovary), C57 (other and unspecified female genital organs) and C76 and C80 (abdomen, pelvis, other ill-defined sites, and unknown primary site). The JRC-ENCR system accepts morphology codes 8441, 8460 and 8471 also for cancers in C54 (corpus uteri), and code 8470 not only for cancers of the female genital tract (C56, C57) but also in C18 (colon) and C25 (pancreas). The result is a marked difference in warnings by the two systems: 585 by IARC and 24 by JRC-ENCR.

The IARC check system devised consistency checks between tumor site and morphology using the data collected in its large database, similar to what Berg did when he devised a system based on morphologic similarities and differences for the recognition of multiple tumors (14). In addition, the IARC checks refer to groups of morphologies that are accepted only for tumors arising in certain organs (tumors with a specific site profile) or that are not allowed in certain organs (tumors with an inverse site profile); there is also a group of morphologies that have no organ specificity and can be assigned to tumors arising in any organ (tumors with no specific site profile) (5). This leads to different choices in generating errors or warnings compared to the JRC-ENCR system. The IARC system only checks morphologies that are normally attributed on the basis of a cytologic/histologic diagnosis, whereas the JRC-ENCR system also performs the opposite check: generic morphology codes (8000, 9590, 9960) with a basis of cytologic/histologic diagnosis.

#### 4.2.2 Staging and follow-up variables

The JRC-ENCR system gives out more warnings related to variables not considered in the IARC system (e.g., TNM stage, *TNM Staging Manual* edition, patient follow-up). Many of the reported warnings are due to incorrect coding of tumor stage or to the combination of a clinical basis of diagnosis and pathologic stage variables. Not all cancer registries can code tumor stage at diagnosis; moreover, the use of incorrect codes related to pathologic and clinical staging is frequent. Minicozzi’s study of the presence and quality of staging at diagnosis in European population-based cancer registries showed that only half of the Italian registries participating in the study were able to provide staging information; particularly case records compiled in an automated manner or directly from pathology laboratory reports were lacking this variable (25).

These checks, along with demographic data, ensure appropriate staging of registered cases, making it possible to study cancer survival and improving the accuracy of the registry’s output. For example, it is unlikely that an advanced-stage neoplasm in the lung or pancreas will grant the patient who carries it a long survival time (26).

#### 4.2.3 Behavior and stage

The unlikely combination of a tumor’s behavior code and its registered stage (e.g., infiltrating carcinoma with behavior code/3 and *in situ* stage, pTis) (170 warnings) will lead the registry to review the case because of a suspected registration error. A possible scenario, on the other hand, is that of an *in situ* neoplasm developing aggressive behavior over time and ultimately generating metastases (27).

#### 4.2.4 Histology and grade

Both the JRC-ENCR and IARC systems flag issues related to the incorrect combination of histology and tumor grade. Grade refers to differentiation in solid tumors (codes 1, 2, 3, 4, 9): it is a measurement of how closely the tumor cells resemble the parent tissue (organ of origin) (See **Supplementary Materials**). Well-differentiated tumor cells (grade 1) closely resemble the tissue from the organ of origin. Poorly differentiated (grade 3) and undifferentiated (grade 4) tumor cells are disorganized and abnormal looking. Codes 5, 6, 7 and 8 are cell indicators, because they describe the lineage or phenotype of the cell and are used only for hematopoietic and lymphoid neoplasms; code 9 indicates cell type not determined, not stated, or not applicable (13). Both systems follow a specific routine to identify incorrect or missing combinations to be flagged for revision (9).

The systems check the morphology codes of solid tumors requiring a specific grade (e.g., undifferentiated sarcoma 8805/3 with grade 4). The JRC-ENCR system will flag the combination of grades 5-8 and morphology codes outside the 9590-9992 range (hematopoietic system codes). The IARC system also performs the opposite check, flagging grade codes greater than or equal to 1 and less than or equal to 4 in combination with histology codes greater than or equal to 9590. Moreover, the IARC system flags more cases because it requires a specific grade for many hematopoietic neoplasms and does not accept the value 9 (not specified); for example, all B-cell lymphomas should have grade 6.

#### 4.2.5 Histology and age

Another issue flagged by the systems concerns inappropriate combinations of tumor morphology and patient age at diagnosis, e.g., 9945 (chronic myelomonocytic leukemia) or 9876 (atypical chronic myeloid leukemia) for age less than 30 years; cancer site C51-C52 (vulva and vagina) for age less than 20 years, or cancer site C60 (penis) for age less than 30 years. Burkitt lymphoma (code 9687) is expected to be diagnosed in children aged less than 14 years, but the registries use the same morphology code for Burkitt-like lymphoma. The distinction between Burkitt and Burkitt-like lymphoma is morphologic: tumor cells in Burkitt-like lymphoma are slightly larger, with more nuclear variability and increased nucleolar prominence. This tumor may arise in patients with a median age of 47 years, but the use of this code in patients aged more than 14 years generated many warnings (28).

The JRC-ENCR system also takes into account patient age for some morphologies. For example, the system accepts basis of diagnosis 2 (clinical) for 8960 (Wilms tumor, nephroblastoma) at age 0-8 years, or basis of diagnosis 4 (specific tumor markers) for 9732 (multiple myeloma) at ages over 40 years; the IARC system does not consider age in these cases but only morphology.

#### 4.2.6 Basis of diagnosis and morphology

The two systems differ in how they treat the variables “basis of diagnosis” and “morphology”. The JRC-ENCR system marks a larger number of cases, because it flags some morphologies that are accepted by the IARC system with a clinical basis of diagnosis (1 or 2), e.g., 8170 (hepatocellular carcinoma), 9732 (multiple myeloma) and 9761 (Waldenstrom globulinemia). Moreover, for cases with death certificate only (DCO) as the basis of diagnosis, the JRC-ENCR system accepts morphologies that can be identified from the underlying cause of death code (ICD-10), while the IARC system flags all DCO cases with morphologies different from those accepted even without microscopic verification.

Another difference between the two systems is related to morphology code 8720 (melanoma): in the absence of histologic or cytologic examination the IARC system accepts only cases arising in C44 (skin) or C69 (eye and adnexa), whereas the JRC-ENCR system accepts melanoma arising at any site.

The large number of warnings detected by the systems is also due to the increased use of electronic health data. In hospital discharge records some cancer codes from the ICD-9-CM classification contain morphologic information (e.g., Hodgkin lymphoma, melanoma, myeloid leukemia, lymphoid leukemia, mycosis fungoides, non-Hodgkin lymphoma): to make incidence calculations, the registries use these codes associated with a clinical basis of diagnosis, but this is not accepted by the JRC-ENCR system in combination with such specific morphology.

The same applies to *in situ* neoplasms: these require a histologic basis of diagnosis, but the information may have come from hospital discharge records, where some tumor sites are labeled with a specific code when they exhibit *in situ* behavior (e.g., in ICD-9-CM, 233.0, carcinoma *in situ* of breast or 233.7, carcinoma *in situ* of bladder).

The JRC-ENCR system performs an additional check for basis of diagnosis 6 (histology of a metastasis): it considers it unlikely that a lymphoma or leukemia diagnosis is based on a metastasis (W-BDMU) (811 cases), whereas a bone marrow aspirate can be used as the basis of diagnosis for lymphomas.

#### 4.2.7 Sex and histology

With regard to sex/histology consistency checks, IARC warnings are mostly due to unacceptable combinations, such as typical ovarian histology in cancer arising in C25 (pancreas) in a male patients while the JRC-ENCR system flags only cases in which ovarian morphology is not allowed in C25, e.g., 8471 (papillary mucinous cystadenocarcinoma).

## 5 Conclusion

The IARC/IACR CHECK program, intended for cancer registries worldwide, utilizes a less demanding checking system that is easy to use for all registries. At present its checking routine for histology requires updating with the new morphology codes included in the second revision of ICD-O-3. The JRC-JRC-ENCR quality check software carries out a number of additional checks compared to IARC. For this reason, it would be advisable to use both systems for data quality control, since they provide checks on different groups of variables (stage, follow-up) or on the same variables but with different modalities.

Finally, periodic checks are useful for identifying issues that inevitably arise when working with data. However, it is important to find the right balance between the need to maintain high standards of data quality – otherwise the data are useless – and the workability of such systems in the daily routine of the cancer registry.

## Data availability statement

The data analyzed in this study is subject to the following licenses/restrictions: The data on cancer cases used in this study were provided by cancer registries affiliated with AIRTUM and cannot be made freely available. Requests to access these datasets should be directed to not available.

## Author contributions

(I) Conception and design: GT, PC. (II) Revising the work critically for important intellectual content: GT, PC, VP, SF, AT, GB, MV. (III) Collection and assembly of data: SF, VP, AT, GB. (IV) Data analysis and interpretation: GT, VP, SF, AT, GB, MV. (V) Manuscript writing: GT and VP. (VI) Final approval of manuscript: GT, PC, VP, SF, AT, GB, MV. (VII) Agreement to be accountable for all aspects of the work in ensuring that questions related to the accuracy or integrity of any part of the work are appropriately investigated and resolved: GT, PC, VP, SF, AT, GB, MV. All authors contributed to the article and approved the submitted version.

## Group members of Collaborative Working Group

Lorenza Boschetti, Stefano Marguati (Pavia CR); Giuseppa Candela, Tiziana Scuderi, Giovanna Fantaci (Trapani-Agrigento CR); Maurizio Zarcone, Sergio Mazzola, Barbara Ravazzolo (Palermo CR); Daniela Piras, Elisa Conca, Maria Rotella (Sassari CR); Pasquala Pinna, Roberta Bosu, Luisa Canu (Nuoro CR); Diego Serraino, Luigino Dal Maso, Federica Toffolutti (Friuli Venezia Giulia CR and CRO Aviano); Antonella Puppo, Claudia Casella, Enza Mariani (Genova CR); Serena Maria Curti, Salvatore Bongiorno, Claudio Trentin (Valle d’Aosta CR); Margherita Ferrante, Fiorella Paderni, Andrea Benedetto (Catania-Messina-Enna CR); Roberto Vito Rizzello, Maria Adalgisa Gentilini, Mariangela Pedron (Trento CR); Anna Clara Fanetti, Ivan Cometti, Paola Giumelli (Sondrio CR); Rosario Tumino, Francesca Bella, Antonino Colanino Ziino (Siracusa CR); Maria Francesca Vitale, Valerio Ciullo (Campania - Napoli 3 CR); Alessio Gili, Silvia Leite, Fortunato Bianconi (Umbria CR); Fabio Vittadello, Paolo Vian, Michael Mian (Bolzano CR); Giuseppe Cascone, Eugenia Spata, Antonella Ippolito (Ragusa-Caltanissetta CR); Giancarlo D’Orsi (Campania – Napoli 2 CR); Rosanna Ortolani, Valentina Cozza, Lucia De Coppi (Campania – Napoli 1 CR); Rossella Cavallo (Salerno CR); Maddalena Merola, Antonietta Minichino, Nadia Di Pascale (Caserta CR); Anna Rita Citarella (Benevento CR); Francesco D’Amelio (Avellino CR).
