# Supplementary material for: Comparison between two cancer registry quality check systems: functional features and differences in an Italian network of cancer registries dataset
Source: Front Oncol. 2023 May 25;13:1197942. doi: 10.3389/fonc.2023.1197942 (PMC10250004; doi:10.3389/fonc.2023.1197942)
Supplement: Supplementary file 1 [file Table_1.docx]

Supplementary Material

TABLE S1. Edit checks carried out by the IARC/IACR CHECK program: types of errors (A) and warnings (B).

| A) | **Error codes** |
| --- | --- |
|  | Age/Incidence and birth dates |
|  | Sex/Site |
|  | Behavior/Histology |
|  | ICD-O-3 (Morphology not valid) |
|  | ICD-O-3 (Topography not valid) |

| B) | **Warning codes** |
| --- | --- |
|  | Age/Site/Histology |
|  | Basis of diagnosis/Histology |
|  | Behavior/Histology |
|  | Behavior/Site |
|  | Histology/Site |
|  | Sex/Histology |
|  | Grade/Histology |

TABLE S2. Types of errors (A) and warnings (B) reported by the JRC-ENCR quality check sofware.

| A) | **Error codes** | |
| --- | --- | --- |
|  | E-AGEC | Age is invalid or missing, and cannot be calculated |
|  | E-AGED | Date of incidence – Date of birth (calculated in years differs from variable Age by more than one year) |
|  | E-CoDA | Date of birth and date of incidence are not consistent |
|  | E-CoDV | Date of incidence and date of last known vital status are not consistent |
|  | E-DUPL | ID/tumor ID combination is repeated in two or more records |
|  | E-ECOD | ICD edition and cause of death combination are not valid |
|  | E-FORM | Format error |
|  | E-MISS | Value missing |
|  | E-OUTR | Out of range |
|  | E-RECO | Record has the wrong number of fields |
|  | E-SETO | Sex and topography combinations are not valid |

| B) | **Warning codes** | |
| --- | --- | --- |
|  | W-AGMT | Unlikely age and morphology/topography combination |
|  | W-BDMO | Morphology too specific according to basis of diagnosis |
|  | W-BDMS | Morphology not specific enough according to basis of diagnosis |
|  | W-BDMU | Basis of diagnosis and morphology/behavior combination is unlikely |
|  | W-BDpM | Basis of diagnosis and pM combination is not valid |
|  | W-BDpN | Basis of diagnosis and pN combination is not valid |
|  | W-BDpT | Basis of diagnosis and pT combination is not valid |
|  | W-BEGR | Behavior and grade combination is not valid |
|  | W-BTNM | Invalid behavior and TNM combination |
|  | W-EDIM | TNM edition and pM are not consistent |
|  | W-MISS | Value missing |
|  | W-MOBE | Morphology and behavior combinations are not included in ICD-O-3 |
|  | W-MOGR | Morphology and grade combination is unlikely |
|  | W-MOTO | Morphology and topography combination is unlikely |
|  | W-MPMT | Multiple primary tumors |
|  | W-SEMO | Sex and morphology combination is unlikely |
|  | W-TNME | TNM and stage are present, but TNM edition is not valid or missing |
|  | W-TNMM | TNM and stage are present, but the morphology is not included in the TNM |
|  | W-TNMS | TNM and stage are not consistent |
|  | W-UNKN | A variable with no impact on incidence calculations has a missing value |

TABLE S3. Definition of the variable Basis of diagnosis.

| **Basis of diagnosis** | |
| --- | --- |
| 0 | Death certificate only (DCO) |
| 1 | Clinical |
| 2 | Clinical investigation |
| 4 | Specific tumor markers |
| 5 | Cytology |
| 6 | Histology of a metastasis |
| 7 | Histology of a primary tumor |
| 9* | Unknown |

*Warning for value = 9

TABLE S4. Definition of the variable Tumor grade.

| **Grade (ICD-O-3)** | |
| --- | --- |
| 1 | Well differentiated |
| 2 | Moderately differentiated |
| 3 | Poorly differentiated |
| 4 | Undifferentiated, anaplastic |
| 5 | T-cell; T-precursor |
| 6 | B cell; Pre-B; B-precursor |
| 7 | Null cell; Non T-non B |
| 8 | NK (natural killer) cell |
| 9 | Unknown |

TABLE S5. Topography codes of ICD-O-3.

| **Code** | **Description** |
| --- | --- |
| C00.0 | External upper lip |
| C00.1 | External lower lip |
| C00.2 | External lip NOS |
| C00.3 | Mucosa of upper lip |
| C00.4 | Mucosa of lower lip |
| C00.5 | Mucosa of lip NOS |
| C00.6 | Commissure of lip |
| C00.8 | Overlapping lesion of lip |
| C00.9 | Lip NOS |
| C01.9 | Base of tongue NOS |
| C02.0 | Dorsal surface of tongue NOS |
| C02.1 | Border of tongue |
| C02.2 | Ventral surface of tongue NOS |
| C02.3 | Anterior 2/3 of tongue NOS |
| C02.4 | Lingual tonsil |
| C02.8 | Overlapping lesion of tongue |
| C02.9 | Tongue NOS |
| C03.0 | Upper gum |
| C03.1 | Lower gum |
| C03.9 | Gum NOS |
| C04.0 | Anterior floor of mouth |
| C04.1 | Lateral floor of mouth |
| C04.8 | Overlapping lesion of floor of mouth |
| C04.9 | Floor of mouth NOS |
| C05.0 | Hard palate |
| C05.1 | Soft palate NOS |
| C05.2 | Uvula |
| C05.8 | Overlapping lesion of palate |
| C05.9 | Palate NOS |
| C06.0 | Cheek mucosa |
| C06.1 | Vestibule of mouth |
| C06.2 | Retromolar area |
| C06.8 | Overlapping lesion of other and unspecified parts of mouth |
| C06.9 | Mouth NOS |
| C07.9 | Parotid gland |
| C08.0 | Submandibular gland |
| C08.1 | Sublingual gland |
| C08.8 | Overlapping lesion of major salivary gland |
| C08.9 | Major salivary gland NOS |
| C09.0 | Tonsillar fossa |
| C09.1 | Tonsillar pillar |
| C09.8 | Overlapping lesion of tonsil |
| C09.9 | Tonsil NOS |
| C10.0 | Vallecula |
| C10.1 | Anterior surface of epiglottis |
| C10.2 | Lateral wall of oropharynx |
| C10.3 | Posterior wall of oropharynx |
| C10.4 | Branchial cleft |
| C10.8 | Overlapping lesion of oropharynx |
| C10.9 | Oropharynx NOS |
| C11.0 | Superior wall of nasopharynx |
| C11.1 | Posterior wall of nasopharynx |
| C11.2 | Lateral wall of nasopharynx |
| C11.3 | Anterior wall of nasopharynx |
| C11.8 | Overlapping lesion of nasopharynx |
| C11.9 | Nasopharynx NOS |
| C12.9 | Pyriform sinus |
| C13.0 | Postcricoid region |
| C13.1 | Aryepiglottic fold |
| C13.2 | Posterior wall of hypopharynx |
| C13.8 | Overlapping lesion of hypopharynx |
| C13.9 | Laryngopharynx, hypopharynx NOS |
| C14.0 | Pharynx NOS |
| C14.2 | Waldeyer's ring NOS |
| C14.8 | Overlapping lesion of lip, oral cavity and pharynx |
| C15.0 | Cervical esophagus |
| C15.1 | Thoracic esophagus |
| C15.2 | Abdominal esophagus |
| C15.3 | Upper third of esophagus |
| C15.4 | Middle third of esophagus |
| C15.5 | Lower third of esophagus |
| C15.8 | Overlapping lesion of esophagus |
| C15.9 | Esophagus NOS |
| C16.0 | Cardia NOS |
| C16.1 | Fundus of stomach |
| C16.2 | Body of stomach |
| C16.3 | Gastric antrum |
| C16.4 | Pylorus |
| C16.5 | Lesser curvature of stomach NOS |
| C16.6 | Greater curvature of stomach NOS |
| C16.8 | Overlapping lesion of stomach |
| C16.9 | Stomach NOS |
| C17.0 | Duodenum |
| C17.1 | Jejunum |
| C17.2 | Ileum |
| C17.3 | Meckel's diverticulum |
| C17.8 | Overlapping lesion of small intestine |
| C17.9 | Small intestine |
| C18.0 | Cecum |
| C18.1 | Appendix |
| C18.2 | Ascending colon |
| C18.3 | Hepatic flexure of colon |
| C18.4 | Transverse colon |
| C18.5 | Splenic flexure of colon |
| C18.6 | Descending colon |
| C18.7 | Sigmoid colon |
| C18.8 | Overlapping lesion of colon |
| C18.9 | Colon NOS |
| C19.9 | Rectosigmoid junction |
| C20.9 | Rectum NOS |
| C21.0 | Anus NOS |
| C21.1 | Anal canal |
| C21.2 | Cloacogenic zone |
| C21.8 | Overlapping lesion of rectum, anus and anal canal |
| C22.0 | Liver |
| C22.1 | Intrahepatic bile duct |
| C23.9 | Gallbladder |
| C24.0 | Extrahepatic bile duct |
| C24.1 | Ampulla of Vater |
| C24.8 | Overlapping lesion of biliary tract |
| C24.9 | Biliary tract NOS |
| C25.0 | Head of pancreas |
| C25.1 | Body of pancreas |
| C25.2 | Tail of pancreas |
| C25.3 | Pancreatic duct |
| C25.4 | Islets of Langerhans |
| C25.7 | Other specified parts of pancreas |
| C25.8 | Overlapping lesion of pancreas |
| C25.9 | Pancreas NOS |
| C26.0 | Intestinal tract NOS |
| C26.8 | Overlapping lesion of digestive system |
| C26.9 | Gastrointestinal tract NOS |
| C30.0 | Nasal cavity |
| C30.1 | Middle ear |
| C31.0 | Maxillary sinus |
| C31.1 | Ethmoid sinus |
| C31.2 | Frontal sinus |
| C31.3 | Sphenoid sinus |
| C31.8 | Overlapping lesion of accessory sinuses |
| C31.9 | Accessory sinus NOS |
| C32.0 | Glottis |
| C32.1 | Supraglottis |
| C32.2 | Subglottis |
| C32.3 | Laryngeal cartilage |
| C32.8 | Overlapping lesion of larynx |
| C32.9 | Larynx NOS |
| C33.9 | Trachea |
| C34.0 | Main bronchus |
| C34.1 | Upper lobe lung |
| C34.2 | Middle lobe lung |
| C34.3 | Lower lobe lung |
| C34.8 | Overlapping lesion of lung |
| C34.9 | Lung NOS |
| C37.9 | Thymus |
| C38.0 | Heart |
| C38.1 | Anterior mediastinum |
| C38.2 | Posterior mediastinum |
| C38.3 | Mediastinum NOS |
| C38.4 | Pleura NOS |
| C38.8 | Overlapping lesion of heart, mediastinum and pleura |
| C39.0 | Upper respiratory tract |
| C39.8 | Overlapping lesion of respiratory system |
| C39.9 | Ill-defined sites within respiratory system |
| C40.0 | Long bones of upper limb, scapula and associated joints |
| C40.1 | Short bones of upper limb |
| C40.2 | Long bones of lower limb |
| C40.3 | Short bones of lower limb |
| C40.8 | Overlapping lesion of bones, joints and articular cartilage of limbs |
| C40.9 | Bone of limb NOS |
| C41.0 | Bones of skull and face |
| C41.1 | Mandible |
| C41.2 | Vertebral column |
| C41.3 | Rib, sternum, clavicle and associated joints |
| C41.4 | Pelvic bones, sacrum, coccyx and associated joints |
| C41.8 | Overlapping lesion of bones |
| C41.9 | Bone NOS |
| C42.0 | Blood |
| C42.1 | Bone marrow |
| C42.2 | Spleen |
| C42.3 | Reticuloendothelial system NOS |
| C42.4 | Hematopoietic system NOS |
| C44.0 | Skin of lip NOS |
| C44.1 | Eyelid |
| C44.2 | External ear |
| C44.3 | Skin of other and unspecified parts of face |
| C44.4 | Skin of scalp and neck |
| C44.5 | Skin of trunk |
| C44.6 | Skin of upper limb and shoulder |
| C44.7 | Skin of lower limb and hip |
| C44.8 | Overlapping lesion of skin |
| C44.9 | Skin NOS |
| C47.0 | Peripheral nerves and autonomic nervous system of head, face and neck |
| C47.1 | Peripheral nerves and autonomic nervous system of upper limb and shoulder |
| C47.2 | Peripheral nerves and autonomic nervous system of lower limb and hip |
| C47.3 | Peripheral nerves and autonomic nervous system of thorax |
| C47.4 | Peripheral nerves and autonomic nervous system of abdomen |
| C47.5 | Peripheral nerves and autonomic nervous system of pelvis |
| C47.6 | Peripheral nerves and autonomic nervous system of trunk |
| C47.8 | Overlapping lesion of peripheral nerves and autonomic nervous system |
| C47.9 | Autonomic nervous system NOS |
| C48.0 | Retroperitoneum |
| C48.1 | Specified parts of peritoneum |
| C48.2 | Peritoneum NOS |
| C48.8 | Overlapping lesion of retroperitoneum and peritoneum |
| C49.0 | Soft tissues of head, face and neck |
| C49.1 | Soft tissues of upper limb and shoulder |
| C49.2 | Soft tissues of lower limb and hip |
| C49.3 | Soft tissues of thorax |
| C49.4 | Soft tissues of abdomen |
| C49.5 | Soft tissues of pelvis |
| C49.6 | Soft tissues of trunk |
| C49.8 | Overlapping lesion of soft tissues |
| C49.9 | Other soft tissues |
| C50.0 | Nipple |
| C50.1 | Central portion of breast |
| C50.2 | Upper-inner quadrant of breast |
| C50.3 | Lower-inner quadrant of breast |
| C50.4 | Upper-outer quadrant of breast |
| C50.5 | Lower-outer quadrant of breast |
| C50.6 | Axillary tail of breast |
| C50.8 | Overlapping lesion of breast |
| C50.9 | Breast NOS |
| C51.0 | Labium majus |
| C51.1 | Labium minus |
| C51.2 | Clitoris |
| C51.8 | Overlapping lesion of vulva |
| C51.9 | Vulva NOS |
| C52.9 | Vagina NOS |
| C53.0 | Endocervix |
| C53.1 | Exocervix |
| C53.8 | Overlapping lesion of cervix uteri |
| C53.9 | Cervix uteri |
| C54.0 | Isthmus uteri |
| C54.1 | Endometrium |
| C54.2 | Myometrium |
| C54.3 | Fundus uteri |
| C54.8 | Overlapping lesion of corpus uteri |
| C54.9 | Corpus uteri |
| C55.9 | Uterus NOS |
| C56.9 | Ovary |
| C57.0 | Fallopian tube |
| C57.1 | Broad ligament |
| C57.2 | Round ligament |
| C57.3 | Parametrium |
| C57.4 | Uterine adnexa |
| C57.7 | Other parts of female genital organs |
| C57.8 | Overlapping lesion of female genital organs |
| C57.9 | Female genital tract NOS |
| C58.9 | Placenta |
| C60.0 | Prepuce |
| C60.1 | Glans penis |
| C60.2 | Body of penis |
| C60.8 | Overlapping lesion of penis |
| C60.9 | Penis NOS |
| C61.9 | Prostate gland |
| C62.0 | Undescended testis |
| C62.1 | Descended testis |
| C62.9 | Testis NOS |
| C63.0 | Epididymis |
| C63.1 | Spermatic cord |
| C63.2 | Scrotum NOS |
| C63.7 | Other parts of male genital organs |
| C63.8 | Overlapping lesion of male genital organs |
| C63.9 | Male genital organs NOS |
| C64.9 | Kidney NOS |
| C65.9 | Renal pelvis |
| C66.9 | Ureter |
| C67.0 | Trigone of urinary bladder |
| C67.1 | Dome of urinary bladder |
| C67.2 | Lateral wall of urinary bladder |
| C67.3 | Anterior wall of urinary bladder |
| C67.4 | Posterior wall of urinary bladder |
| C67.5 | Bladder neck |
| C67.6 | Ureteric orifice |
| C67.7 | Urachus |
| C67.8 | Overlapping lesion of bladder |
| C67.9 | Urinary bladder NOS |
| C68.0 | Urethra |
| C68.1 | Paraurethral gland |
| C68.8 | Overlapping lesion of urinary organs |
| C68.9 | Urinary system NOS |
| C69.0 | Conjunctiva |
| C69.1 | Cornea NOS |
| C69.2 | Retina |
| C69.3 | Choroid |
| C69.4 | Ciliary body |
| C69.5 | Lacrimal gland NOS |
| C69.6 | Orbit NOS |
| C69.8 | Overlapping lesion of eye adnexa |
| C69.9 | Eye NOS |
| C70.0 | Cerebral meninges |
| C70.1 | Spinal meninges |
| C70.9 | Meninges NOS |
| C71.0 | Cerebrum |
| C71.1 | Frontal lobe |
| C71.2 | Temporal lobe |
| C71.3 | Parietal lobe |
| C71.4 | Occipital lobe |
| C71.5 | Ventricle NOS |
| C71.6 | Cerebellum NOS |
| C71.7 | Brain stem |
| C71.8 | Overlapping lesion of brain |
| C71.9 | Brain NOS |
| C72.0 | Spinal cord |
| C72.1 | Cauda equina |
| C72.2 | Olfactory nerve |
| C72.3 | Optic nerve |
| C72.4 | Acoustic nerve |
| C72.5 | Cranial nerve |
| C72.8 | Overlapping lesion of brain and CNS |
| C72.9 | Nervous system NOS |
| C73.9 | Thyroid gland |
| C74.0 | Cortex of adrenal gland |
| C74.1 | Medulla of adrenal gland |
| C74.9 | Adrenal gland NOS |
| C75.0 | Parathyroid gland |
| C75.1 | Pituitary gland |
| C75.2 | Craniopharyngeal duct |
| C75.3 | Pineal gland |
| C75.4 | Carotid body |
| C75.5 | Aortic body and other paraganglia |
| C75.8 | Overlapping lesion of endocrine glands |
| C75.9 | Endocrine gland NOS |
| C76.0 | Head, face or neck NOS |
| C76.1 | Thorax NOS |
| C76.2 | Abdomen NOS |
| C76.3 | Pelvis NOS |
| C76.4 | Upper limb NOS |
| C76.5 | Lower limb NOS |
| C76.7 | Other ill-defined sites |
| C76.8 | Overlapping lesion of ill-defined sites |
| C77.0 | Lymph nodes of head, face and neck |
| C77.1 | Intrathoracic lymph nodes |
| C77.2 | Intra-abdominal lymph nodes |
| C77.3 | Lymph nodes of axilla or arm |
| C77.4 | Lymph nodes of inguinal region or leg |
| C77.5 | Pelvic lymph nodes |
| C77.8 | Lymph nodes of multiple regions |
| C77.9 | Lymph node NOS |
| C80.9 | Unknown primary site |
